# HIV Testing and Sexual Health Among Black African Men and Women in London, United Kingdom

**DOI:** 10.1001/jamanetworkopen.2019.0864

**Published:** 2019-03-22

**Authors:** Ibidun Fakoya, Louise Logan, Winnie Ssanyu-Sseruma, Alison Howarth, Gary Murphy, Anne M. Johnson, Anthony Nardone, Alison J. Rodger, Fiona Burns

**Affiliations:** 1 Institute for Global Health, University College London, London, United Kingdom; 2 HIV and STIs Department, Public Health England, London, United Kingdom; 3 Independent Consultant, United Kingdom; 4 Royal Free London NHS Foundation Trust, London, United Kingdom

## Abstract

**Importance:**

Black African adults are disproportionately affected by HIV in the United Kingdom. Many within this population acquire HIV after migration or are diagnosed late. Data are needed to inform targeted interventions to increase HIV testing and prevention in this population.

**Objective:**

To inform future HIV prevention strategies by estimating diagnosed and undiagnosed HIV infection and measuring changes in HIV testing rates in black African adults in London, United Kingdom.

**Design, Setting, and Participants:**

This cross-sectional study used a self-completed survey conducted from September 20 to December 3, 2016. Questionnaires were linked to an optional, anonymous oral fluid HIV test and compared with data from a previous survey (2004). Respondents were recruited from social and commercial venues frequented by black African adults in London. Of 2531 individuals approached in 63 venues, 752 agreed to participate. Data were analyzed initially in March 2017 (as part of internal reporting) and again in August 2018.

**Main Outcomes and Measures:**

Self-reported HIV testing within the past 5 years; diagnosed and undiagnosed HIV prevalence. Logistic regression examined factors associated with HIV testing by sex.

**Results:**

In total, 292 women (median [interquartile range] age, 31 [25.0-41.5] years) and 312 men (median [interquartile range] age, 35 [25.0-41.5] years) were included in the analysis. More than half of men (159 [51.0%]) and women (154 [52.7%]) had been tested for HIV in the past 5 years. In multivariable analysis, HIV testing was associated with a range of factors in both sexes, including health service attendance, time in the United Kingdom, and sexually transmitted infection diagnosis. Increases in HIV testing in the past 5 years were observed between 2004 and 2016 for both sexes. In the 2016 sample, 219 of 235 women (93.2%) and 206 of 228 men (90.4%) tested HIV negative. Among those testing positive, 56.3% of women (9 of 16) and 40.9% of men (9 of 22) self-reported as HIV negative or untested, indicating they were living with undiagnosed HIV. A fifth of women (20.7%) and 25.0% of men reported condomless last sex with a partner of different or unknown HIV status in the past year.

**Conclusions and Relevance:**

Despite efforts to increase HIV testing, uptake in black African communities in London remains modest. This study identified a large fraction of undiagnosed infection—greater than other at-risk populations—suggesting that the prevention and care needs of this group are not adequately met.

## Key Points

**Question:**

What proportion of black African adults attending social and commercial venues in London, United Kingdom, have been tested for HIV in the past 5 years and what proportion are living with HIV—both diagnosed and undiagnosed?

**Findings:**

In this cross-sectional study of 604 respondents, 51.0% of men and 52.7% of women had undergone HIV testing in the past 5 years. Of respondents who provided an oral fluid sample for anonymized HIV testing, 93.2% of women and 90.4% of men were HIV negative; among those testing positive, 56.3% of women and 40.9% of men self-reported as HIV negative or untested, indicating they were living with undiagnosed HIV.

**Meaning:**

Despite ongoing campaigns to increase HIV testing in African communities in London, the fraction living with undiagnosed HIV remains unacceptably high; therefore, testing coverage must be enhanced to meet the HIV prevention needs of this group.

## Teaser

This cross-sectional study investigates the proportion of black African adults in London, United Kingdom, who have been tested for HIV in the past 5 years and the proportion living with diagnosed and undiagnosed HIV.

## Introduction

In the United Kingdom, black African communities are disproportionately represented in the HIV epidemic. In England in 2016, 13.0% of more than 5000 new HIV diagnoses and almost one-quarter (22 100 of 89 400) of people living with HIV (both diagnosed and undiagnosed) were heterosexual black African men and women.^1^ An estimated 2.3% of black African heterosexual men and 3.4% of black African heterosexual women are living with HIV in England.^1^ This contrasts with an estimated prevalence of 0.001% in the general heterosexual adult population.^1^ In London, United Kingdom (where most black African communities live),^2^ an estimated 12 300 black African heterosexual individuals were living with HIV in 2016; an estimated 12% of HIV-positive black African men and 4% of HIV-positive black African women were undiagnosed.^1^

Late diagnosis of HIV (CD4 lymphocyte count <350 cells/μL or an AIDS-defining illness within 3 months of diagnosis) is associated with poorer individual health outcomes and increased population transmission of infection^3^ and is a problem for black African communities in the United Kingdom. In London in 2016, 61% of black African men and 52% of black African women were diagnosed late, compared with 48% of white heterosexual men, 42% of white heterosexual women, and 24% of gay or bisexual men.^1^

To tackle the HIV epidemic in the black African community in the United Kingdom, a better understanding of this population’s HIV prevention needs is required. An estimated 33% to 45% of HIV infections among black African individuals were acquired after migration to the United Kingdom (although it is unclear how travel to and from Africa affects these estimates).^4,5^ Qualitative studies suggest that African migrants do not realize that the risk of HIV acquisition continues after migration.^6,7,8^ Although HIV and sexually transmitted infection (STI) risk are higher in people of black African ethnicity, individual behaviors do not wholly explain this risk, which is affected by sexual network factors independent of individual behavior.^9,10^

High levels of postmigration HIV acquisition and high numbers of late diagnoses indicate that policy makers and program managers should invest additional attention and resources in improving primary prevention programs, which are underpinned by increased access to testing. This is particularly true if black African communities living in the United Kingdom are to benefit from the World Health Organization’s test and treat initiatives, with the resultant reductions in people living with undiagnosed HIV, community viral load, and HIV incidence.^11^ Therefore, data are needed to develop differentiated HIV testing programs tailored to the specific needs of black African communities in the United Kingdom.^12^

It has been several years since the barriers to HIV testing and prevention among black African communities in the United Kingdom were explored.^13,14^ A 2009 community-based study estimated that 42% of black African individuals in England had been tested within the past 5 years.^15^ While this percentage is higher than the estimated 11% to 15% UK general population HIV testing rate,^16^ it is low compared with the 60% to 72% of men who have sex with men in England (another disproportionately affected population^1^) who reported testing in the past year.^17,18,19^

Two community-based studies called *Mayisha* (the Swahili word for *lifestyles*) have examined the HIV-related sexual risks, attitudes, and behaviors of black African individuals living in England.^20,21^ The surveys demonstrated the feasibility and acceptability of collecting sensitive information and (for one survey) biological samples from African communities in England. Mayisha (1999) recruited 748 black African respondents in North London; 34% had previously been tested for HIV.^20^ Mayisha II (2004) recruited 1359 respondents in London, Luton, and the West Midlands; 47.0% of women and 37.6% of men reported an HIV test within the past 5 years.^21^

This article presents findings from the third Mayisha study, Mayisha 2016. We report on factors associated with HIV testing among black African adults living in London and compare HIV testing rates with those reported in 2004. We also provide estimates of condomless sex, STIs, knowledge and use of pre-exposure prophylaxis (PrEP), HIV prevalence, and the proportion of individuals living with undiagnosed HIV.

## Methods

### Study Design

This cross-sectional study used a self-completed questionnaire with optional oral fluid collection from respondents recruited in social and commercial venues frequented by black African adults living and working in London. Participants provided oral informed consent to take part in the study, which received ethical approval from the University College London Research Ethics Committee.

### Social Mapping, Setting, and Sampling

Respondents were recruited from 63 venues. Suitable venues for recruitment were identified through a social mapping exercise as follows. We used the 33 municipal areas (boroughs) and the 649 subunits of boroughs (wards) within Greater London as the initial sampling frame. Census data (2011)^2^ were used to identify the 20 boroughs with the largest number of black African residents. The 6 boroughs with the highest proportion or number of black African residents were automatically included in the sample. Four from the remaining 14 boroughs were selected using the RANDBETWEEN randomization function in Microsoft Excel (Microsoft Corp). Within those 10 boroughs (Greenwich, Southwark, Lambeth, Barking and Dagenham, Lewisham, Barnet, Wandsworth, Merton, Hackney, and Newham), the 5 most populous and 5 randomly selected wards were included.

All venues with a valid premises license (required for selling alcohol or serving hot food between 11 pm and 5 am or providing entertainment) within the chosen wards were identified using local authority registers (195 venues). We identified additional venues through consultation with community-based organizations that formed a community advisory group for the study (29 venues); area visits by researchers (141 venues); an online form circulated to advisory group and researcher contacts (129 venues); and web searches for one-off community events (eg, Black History Month lectures) (130 venues). All venues were contacted by email, telephone, or in person; 241 venues responded and 103 venues agreed to participate.

### Target Population and Inclusion Criteria

To be included in the study respondents had to (1) be aged 18 years or older, (2) be able to give informed consent, and (3) self-identify as Black African or have been born in Africa and also identify as Black Other or Black British.

### Survey Instruments

#### Questionnaire

The questionnaire was designed for rapid self-completion and respondents were asked to provide an oral fluid sample for anonymous HIV testing, the results of which were linked to behavioral data. The questionnaire was based on 2 previous Mayisha surveys^20,21^ and included items about sociodemographic characteristics, health service use, HIV testing history, and recent sexual behavior. New items were included to capture PrEP use and awareness of a local sexual health campaign (conducted by the London HIV Prevention Programme in summer 2016).^22^ The questionnaire was available in English and French.

#### Validation of Device for Oral Fluid Testing for HIV

Respondents who completed the questionnaire were asked to provide an oral fluid sample using the Intercept i2 (Orasure Technologies*)* collection device. The device was validated in June 2016 to ensure that Intercept i2 was compatible for use with the third-generation IgG antibody capture enzyme-linked immunosorbent assay (GACELISA); overall sensitivity was 93%, and no false-positives were detected (eAppendix and eTables 1-3 in the Supplement).

### Data Collection and Consent

Respondents were recruited by trained community fieldworkers who visited venues at predetermined dates and times between September 20 and December 3, 2016. Fieldworkers approached potential respondents, explained the purpose of the study, and established whether individuals met the inclusion criteria. To preserve anonymity, no personal identifiable data were collected, only verbal consent was required, and provision of the oral fluid specimen was optional. Fieldworkers explained that the sample was anonymous and respondents would not receive the test results. Written details of how to obtain a subsequent named HIV test were given to all respondents to encourage uptake of HIV testing outside the study. Respondents were given an information sheet and a £5 high street voucher. Completed questionnaires and oral fluid samples were returned to fieldworkers in a sealed, tamper-proof envelope to maintain confidentiality. Individuals younger than 18 years or apparently under the influence of alcohol or other drugs were not approached or offered a questionnaire. Some individuals offered the questionnaire subsequently reported their age as younger than 18 years and were consequently excluded from analysis. Fieldworkers recorded the venue where respondents were recruited as well as information on the number of refusals.

### Oral Fluid Sample Collection and Testing

Respondents self-administered an Intercept i2 oral fluid collection device under the supervision of fieldworkers. All specimens were transported to the National Infections Service in London within 7 days of collection and processed within 21 days. Specimens were tested using the in-house GACELISA assay; specimens giving an optical density/cutoff (OD/CO) ratio greater than 1.0 were presumed reactive, repeat tested, and confirmed by Western blot (HIV blot 2.2; Genelabs). All specimens were tested for total IgG concentration to ensure specimen quality, and those with IgG levels less than 0.02 mg/dL (to convert to grams per liter, multiply by 0.01) were excluded from analysis.

### Outcomes and Variables

The primary outcome was self-reported HIV testing in the past 5 years. Secondary outcomes were HIV status, condomless last sex, sex with a partner of a different or unknown HIV status, and previous STI diagnosis. Participants’ HIV status was determined by oral fluid samples and is reported as HIV negative (respondents who tested negative for anti-HIV antibodies regardless of self-report); HIV positive, previously diagnosed (respondents who self-reported HIV positive and tested HIV positive); and HIV positive, undiagnosed (respondents who self-reported HIV negative or untested but tested HIV positive).

### Sample Size

Previous studies^15,21^ estimated that 42% of the target population had been tested for HIV in the past 5 years (the primary outcome). A sample size of 750 was therefore needed to accurately estimate the primary outcome (with a margin of error of ±5%). The sample size allowed for comparison with the Mayisha II survey^21^ and to detect a change in the primary outcome up to 1.4 times higher or lower in the new survey with 80% or greater power and at a 5% statistical significance level.

### Data Analysis

All data were double-entered onto Microsoft Access 2010 (Microsoft Corp) and analyzed using Stata statistical software version 14.2 (StataCorp LP). Demographic characteristics were compared using Wald χ^2^ for categorical variables and either *t* tests or Mann-Whitney *U* tests for continuous variables. Binary logistic regression was used to analyze associations between demographic and behavioral variables and the primary outcomes. Crude odds ratios (ORs) were calculated for each sex and significant associations in univariate analysis (α = .10) incorporated into multivariable regression modeling using automated backward stepwise selection. Significance testing was set at 2-sided *P* < .05. Associations are reported as OR and adjusted OR (aOR) with 95% confidence intervals.

The prevalence of HIV testing in the past 5 years observed in this study was compared with that observed in the Mayisha II study. Although the studies shared a similar sampling strategy, the previous study had broader geographical and age sampling criteria. In this present analysis, Mayisha II data were limited to those living in London and aged 18 years and older and excluded respondents recruited at health promotion events. Data were analyzed initially in March 2017 (as part of internal reporting) and again in August 2018.

## Results

Fieldworkers approached 2531 individuals, of whom 752 agreed to participate (median [interquartile range] response rate over 122 sessions, 33.3% [14.3%-56.5%]). We excluded 147 respondents because their self-reported ethnicity or age did not match the inclusion criteria (n = 133) or data were missing for more than 90% of questions (n = 14). Most respondents (496 of 604 [82.1%]) included in this present analysis provided an oral fluid specimen, although 33 of 604 specimens (6.7%) were found to have IgG concentrations too low for HIV testing (data not shown).

In total, 292 women and 312 men were included in this analysis. Men in the sample were significantly older than women (median [interquartile range] age, 35 [25.0-41.5] years vs 31 [25.0-41.5] years; *P* = .02) (Table 1). Most respondents were employed (202 of 287 women and 220 of 306 men), and 14.8% of women (34 of 229) and 18.3% of men (50 of 273) born abroad moved to the United Kingdom as refugees or asylum seekers.

**Table 1.** Sociodemographic Characteristics of Respondents to the Mayisha 2016 Survey, Separated by Sex

Characteristics	No./Total No. (%)		*P* Value
	Women	Men	
Respondents	292 (48.3)	312 (51.7)	
Age, median (IQR), y^a^	31 (25.0-41.5)	35 (25.0-41.5)	.02
Time since migration, mean (SD), y^b^^,^^c^	14.5 (7.9)	13.8 (7.9)	.10
Age at migration, mean (SD), y^b^^,^^d^	20.5 (10.1)	22.4 (9.9)	.40
Region of birth^e^			
Western Africa	149/286 (52.1)	151/302 (50.0)	.007
Eastern Africa	49/286 (17.1)	84/302 (27.8)	
Southern Africa	3/286 (1.0)	3/302 (1.0)	
Northern Africa	0/286 (0)	3/302 (1.0)	
Middle Africa	16/286 (5.6)	10/302 (3.3)	
Born outside Africa^f^	69/286 (24.1)	51/302 (16.9)	
Current residence^g^			
North Central London	40/273 (14.7)	28/286 (9.8)	.001
North East London	67/273 (24.5)	98/286 (34.3)	
North West London	25/273 (9.2)	20/286 (7.0)	
South East London	114/273 (41.8)	86/286 (30.1)	
South West London	21/273 (7.7)	40/286 (14.0)	
Outside London	6/273 (2.2)	14/286 (4.9)	
Place of recruitment (n = 533)			
Community center or event	44/257 (17.1)	42/276 (15.2)	<.001
Outdoor event or location	53/257 (20.6)	75/276 (27.2)	
Church or mosque	24/257 (9.3)	5/276 (1.8)	
Club, bar, or restaurant	54/257 (21.0)	83/276 (30.1)	
Hairdresser or barbershop	17/257 (6.6)	54/276 (19.6)	
Retailer or council customer shop	65/257 (25.3)	17/276 (6.2)	
Currently working in paid employment	202/287 (70.4)	220/306 (71.9)	.69
Post-16 education			
None	26/287 (9.1)	33/308 (10.7)	.36
≤2 y	32/287 (11.1)	43/308 (14.0)	
>2 y	176/287 (61.3)	189/308 (61.4)	
Still in full-time education	53/287 (18.5)	43/308 (14.0)	
Relationship status			
Married or cohabiting in United Kingdom	91/280 (32.5)	96/301 (31.9)	.97
In a relationship or married but living apart	62/280 (22.1)	72/301 (23.9)	
Widowed, separated, or divorced	16/280 (5.7)	17/301 (5.6)	
Single	111/280 (39.6)	116/301 (38.5)	
Refugee or asylum seeker (n = 502)	34/229 (14.8)	50/273 (18.3)	.30
Religion (n = 585)			
Christian, all denominations	216/282 (76.6)	211/303 (69.6)	.12
Muslim	55/282 (19.5)	69/303 (22.8)	
Other religion	10/282 (3.5)	18/303 (5.9)	
No religion	1/282 (0.4)	5/303 (1.7)	

Abbreviation: IQR, interquartile range.

^a^ Sample sizes were 255 women and 261 men.

^b^ Excludes UK-born participants.

^c^ Sample sizes were 211 women and 248 men.

^d^ Sample sizes were 177 women and 208 men.

^e^ Based on United Nations Statistic Division area codes.^23^

^f^ Of those born outside Africa, 85.0% were born in the United Kingdom and 11.7% were born in Europe.

^g^ As defined by NHS England.^24^

Most Mayisha 2016 respondents had previously been tested for HIV (203 of 292 women and 206 of 312 men), with 33.2% of women (97 of 292) and 24.4% of men (76 of 312) reporting testing in the past year (Table 2). Testing for HIV within the past 5 years had increased between 2004 and 2016 from 50.8% (131 of 258) to 52.7% (154 of 292) among women (*P* = .65) and from 40.3% (102 of 253) to 51.0% (159 of 312) among men (*P* = .001).

**Table 2.** Self-reported HIV Testing History and HIV Status of Mayisha 2016 and Mayisha II (2004) Survey Respondents

Testing History	No./Total No. (%)			
	Mayisha 2016		Mayisha II (2004)	
	Women	Men	Women	Men
Tested for HIV				
In the last year	97/292 (33.2)	76/312 (24.4)	65/258 (25.2)	43/253 (17.0)
Between 1 and 5 y	57/292 (19.5)	83/312 (26.6)	66/258 (25.6)	59/253 (23.3)
>5 y	49/292 (16.8)	47/312 (15.1)	12/258 (4.7)	20/253 (7.9)
Never	77/292 (26.4)	91/312 (29.2)	112/258 (43.4)	126/253 (49.8)
No response	12/292 (4.1)	15/312 (4.8)	3/258 (1.2)	5/253 (2.0)
Tested for HIV in past 5 y^a^	154/292 (52.7)	159/312 (51.0)	131/258 (50.8)	102/253 (40.3)
HIV status (oral fluid antibody)				
HIV negative	219/235 (93.2)	206/228 (90.4)	178/202 (88.1)	161/189 (85.2)
HIV positive, previously diagnosed	7/235 (3.0)	13/228 (5.7)	8/202 (4.0)	8/189 (4.2)
HIV positive, undiagnosed	9/235 (3.8)	9/228 (3.9)	16/202 (7.9)	20/189 (10.6)
Undiagnosed fraction	9/16 (56.3)	9/22 (40.9)	17/24 (70.8)	20/28 (71.4)

^a^ χ^2^ test comparing prevalence of HIV testing in past 5 years among Mayisha 2016 and Mayisha II (2004) respondents: *P* = .65 for women; *P* = .001 for men.

Most women (219 of 235 [93.2%; 95% CI, 89.2%-98.8%]) and men (206 of 228 [90.4%; 95% CI, 85.8%-93.6%]) who provided an oral fluid specimen tested HIV negative. Among respondents who provided a specimen, 9 of 235 women (3.8%; 95% CI, 2.0%-7.2%) and 9 of 228 men (3.9%; 95% CI, 2.1%-7.4%) self-reported as HIV negative or untested. The undiagnosed fraction was 56.3% (95% CI, 31.4%-78.4%) of HIV-positive women (9 of 16) and 40.9% (95% CI, 22.1%-62.8%) of HIV-positive men (9 of 22). The undiagnosed fraction in Mayisha II was 70.8% (95% CI, 49.4%-85.8%) in women (17 of 24) and 71.4% (95% CI, 51.7%-85.4%) in men (20 of 28). Of the 18 individuals with apparently undiagnosed infection in the Mayisha 2016 sample, 9 self-reported an HIV-negative test in the past 5 years. Anti-HIV antibodies were not detected in 6 of 26 of respondents who self-reported living with diagnosed HIV (Mayisha 2016 sample; data not shown).

Sexual health clinics were the most popular testing location for all respondents (80 of 202 women and 74 of 203 men) (Table 3) followed by general practice (54 of 202 women and 70 of 203 men). Around 1 in 10 women and men reported having had an STI diagnosed in the past 5 years (25 of 275 and 35 of 287, respectively). One in 5 women (50 of 242 [20.7%]) and 1 in 4 men (71 of 284 [25.0%]) reported their most recent sex as condomless sex with a serodifferent partner, a partner with unknown HIV status, or a partner who had never been tested for HIV. Most respondents did not know about PrEP (200 of 272 women and 225 of 294 men) and very small proportions reported knowledge and desire to use PrEP (Table 3).

**Table 3.** Place of HIV Test, Sexual Behavior, and Pre-exposure Prophylaxis of Mayisha 2016 Survey Respondents, Separated by Sex

Factor	No./Total No. (%)	
	Women	Men
Place of last HIV test		
General practice or family doctor	54/202 (26.7)	70/203 (34.5)
Private clinic	15/202 (7.4)	25/203 (12.3)
Sexual health clinic	80/202 (39.6)	74/203 (36.5)
Antenatal	29/202 (14.4)	1/203 (0.5)
Community organization	9/202 (4.5)	13/203 (6.4)
Self-sampling or self-testing	5/202 (2.5)	4/203 (2.0)
Other	10/202 (5.0)	16/203 (7.9)
Previous STI diagnosis		
Ever had an STI	41/275 (14.9)	66/287 (23.0)
STI in the past year	11/275 (4.0)	15/287 (5.2)
STI diagnosis ≤5 y	25/275 (9.1)	35/287 (12.2)
No. of sexual partners in the past 12 mo		
None	82/278 (29.5)	45/299 (15.1)
1	145/278 (52.2)	124/299 (41.5)
2	27/278 (9.7)	51/299 (17.1)
3-4	18/278 (6.5)	40/299 (13.4)
≥5	6/278 (2.2)	39/299 (13.0)
No. of first-time sexual partners in past 12 mo		
None	206/267 (77.2)	163/274 (59.5)
1	41/267 (15.4)	50/274 (18.2)
2	12/267 (4.5)	22/274 (8.0)
3-4	6/267 (2.2)	23/274 (8.4)
≥5	2/267 (0.7)	16/274 (5.8)
Last sexual partner of different ethnicity (disassortative sexual mixing)	45/259 (17.4)	88/298 (29.5)
Used condom at last sex	84/240 (35.0)	124/280 (44.3)
Serosorting at last sex		
Seroconcordant	147/242 (60.7)	151/284 (53.2)
Serodifferent	5/242 (2.1)	8/284 (2.8)
Unknown^a^	90/242 (37.2)	125/284 (44.0)
Condomless last sex with partner of different or unknown HIV status^a^	50/242 (20.7)	71/284 (25.0)
PrEP knowledge and use (n = 566)		
Never heard of PrEP	200/272 (73.5)	225/294 (76.5)
Has heard of PrEP	72/272 (26.5)	69/294 (23.5)
Has heard of and used PrEP	1/272 (0.4)	8/294 (2.7)
Had heard of and would like to use PrEP	7/272 (2.6)	6/294 (2.0)

Abbreviations: PrEP, pre-exposure prophylaxis; STI, sexually transmitted infection.

^a^ Includes all those who have never been tested.

### HIV Testing Among Women

In univariate analysis, HIV testing within the past 5 years was significantly associated with age, years living in the United Kingdom, attending an emergency department in the past year, ever attending a sexual health clinic, and having 1 or more sexual partners in the past year (Table 4). In multivariable analysis, HIV testing remained significantly associated with living in the United Kingdom for 5 years compared with living in the United Kingdom for more than 20 years (aOR, 3.6; 95% CI, 1.0-12.5), attending an emergency department in the past year (aOR, 2.6; 95% CI, 1.0-6.7), ever attending a sexual health clinic (aOR, 2.5; 95% CI, 1.4-4.4), and with increasing numbers of sexual partners in the past 12 months (aOR for 1 partner, 1.8; 95% CI, 1.0-3.5 and aOR for 2 or more partners, 3.2; 95% CI, 1.3-7.6).^23,24^

**Table 4.** Factors Associated With HIV Testing Within Past 5 Years Among Women Participating in the Mayisha 2016 Survey

Factor	Tested in Past 5 y, No./Total No. (%)	OR (95% CI)	*P* Value	aOR (95% CI)^a^	*P* Value
All female respondents^b^	154/270 (57.0)	NA		NA	
Age, y (n = 238)					
18-24	32/70 (45.7)	0.4 (0.2-0.7)	.02	NA	
25-34	54/78 (69.2)	1 [Reference]		NA	
35-44	32/55 (58.2)	0.6 (0.3-1.3)		NA	
≥45	17/35 (48.6)	0.4 (0.2-1.0)		NA	
Region of birth (n = 264)					
Africa	116/196 (59.2)	1 [Reference]	.27	NA	
Other	35/68 (51.5)	0.73 (0.4-1.3)		NA	
Years living in the United Kingdom (n = 240)					
≤5	14/18 (77.8)	2.2 (0.7-6.5)	.008	3.6 (1.0-12.5)	.01
6-10	32/44 (72.7)	1.8 (0.9-3.8)		2.1 (0.9-4.8)	
11-15	22/47 (46.8)	0.7 (0.3-1.3)		0.8 (0.4-1.7)	
16-20	17/43 (39.5)	0.5 (0.2-1.0)		0.5 (0.2-1.2)	
>20	51/88 (58.0)	1 [Reference]		1 [Reference]	
Years of post-16 education (n = 266)					
None	15/23 (65.2)	1.4 (0.6-3.4)	.37	NA	
≤2	14/30 (46.7)	0.6 (0.3-1.4)		NA	
>2 or still in full-time education	123/213 (57.7)	1 [Reference]		NA	
Relationship status (n = 268)					
Married or cohabiting in United Kingdom	39/66 (59.1)	1.2 (0.7-2.3)	.48	NA	
Living apart relationship or marriage	49/79 (62.0)	1.4 (0.8-2.5)		NA	
Single or previously married	66/123 (53.7)	1 [Reference]			
Religion (n = 270)					
Christian, all denominations	121/206 (58.7)	1 [Reference]	.59		
Muslim	27/53 (50.9)	0.7 (0.4-1.3)			
No or other religion	6/11 (54.5)	0.8 (0.2-2.9)			
Sexual behavior (n = 243)					
Gay or bisexual	12/22 (54.5)	0.8 (0.3-2.0)	.64	NA	
Heterosexual	132/221 (59.7)	1 [Reference]		NA	
Attended emergency department or walk-in center in past 12 mo (n = 240)					
No	116/213 (54.5)	1 [Reference]	.10	1 [Reference]	.05
Yes	20/27 (74.1)	2.0 (0.9-4.8)		2.6 (1.0-6.7)	
Health service visit in last 12 mo (n = 270)^c^					
No	40/77 (51.9)	0.7 (0.4-1.3)	.29		
Yes	114/193 (59.1)	1 [Reference]			
Ever attended sexual health clinic (n = 240)					
No	45/105 (42.9)	0.4 (0.2-0.7)	<.001	1 [Reference]	.002
Yes	91/135 (67.4)	1 [Reference]		2.5 (1.4-4.4)	
STI diagnosis (n = 263)					
≤5 y	18/25 (72.0)	2.1 (0.9-5.3)	.16	NA	
>5 y	9/13 (69.2)	1.9 (0.6-6.2)		NA	
Never	123/225 (54.7)	1 [Reference]		NA	
No. of sexual partners in the past 12 mo (n = 240)					
None	28/68 (41.2)	1 [Reference]	.002	1 [Reference]	.03
1	73/125 (58.4)	1.9 (1.1-3.3)		1.8 (1.0-3.5)	
≥2	35/47 (74.5)	3.7 (1.7-8.0)		3.2 (1.3-7.6)	
Used condom at last sex (n = 232)					
No	91/152 (59.9)	1 [Reference]	.70	NA	
Yes	50/80 (62.5)	1.1 (0.6-1.9)		NA	
Living with undiagnosed HIV (n = 215)					
No	119/207 (57.5)	1 [Reference]	.27	NA	
Yes	3/8 (37.5)	0.4 (0.1-1.9)		NA	

Abbreviations: aOR, adjusted odds ratio; NA, not applicable; OR, odds ratio; STI, sexually transmitted infection.

^a^ Adjusted for factors in model selection (length of time in the United Kingdom, attendance at emergency department or walk-in service or attendance at sexual health clinic in past 12 months and number of sexual partners in the past year; n = 241).

^b^ Excludes people diagnosed with HIV longer than 5 years and item nonresponders.

^c^ Antenatal, general practitioner, emergency department, or NHS walk-in center or outpatient appointment.

### HIV Testing Among Men

Among men in the Mayisha 2016 sample, age, region of birth, education, health service attendance in past 12 months, sexual health clinic attendance (ever), previous STI diagnosis, number of sexual partners, and condom use at last sex were all associated with testing within the past 5 years in univariate analysis (Table 5). In multivariable analysis, HIV testing remained significantly associated with age (those aged 25-34 years having the greatest odds of testing), being African born, higher educational attainment, health service attendance in the past 12 months, sexual health clinic attendance (ever), and a previous STI diagnosis (Table 5).

**Table 5.** Factors Associated With Self-reported HIV Testing Within Past 5 Years Among Men Participating Mayisha 2016 Survey

Factor	Tested in Past 5 y, No./Total No. (%)	OR (95% CI)	*P* Value	aOR (95% CI)^a^	*P* Value
All male respondents (n = 287)^b^	159/287 (55.4)	NA		NA	
Age, y (n = 216)					
18-24	14/34 (41.2)	0.4 (0.2-0.8)	.006	0.4 (0.1-1.0)	.03
25-34	54/77 (70.1)	1 [Reference]		1 [Reference]	
35-44	39/60 (65.0)	0.9 (0.5-1.7)		0.6 (0.3-1.4)	
≥45	20/45 (44.4)	0.4 (0.2-0.7)		0.3 (0.1-0.7)	
Region of birth (n = 216)					
Africa	110/178 (61.8)	1 [Reference]	.02	1 [Reference]	.004
Other	17/38 (44.7)	0.5 (0.2-0.9)		0.3 (0.1-0.7)	
Years living in the United Kingdom (n = 265)					
≤5	18/28 (64.3)	1.3 (0.5-3.3)	.70	NA	
6-10	28/55 (50.9)	0.8 (0.4-1.6)		NA	
11-15	40/70 (57.1)	1 [Reference]		NA	
16-20	27/44 (61.4)	1.2 (0.6-2.6)		NA	
>20	36/68 (52.9)	0.8 (0.4-1.7)		NA	
Years of post-16 education (n = 283)					
None	13/31 (41.9)	0.5 (0.2-1.0)	.03	0.6 (0.2-2.4)	.03
<2 y	17/41 (41.5)	0.5 (0.2-0.9)		0.2 (0.1-0.7)	
>2 y or still in full-time education	126/211 (59.7)	1 [Reference]		1 [Reference]	
Relationship status (n = 282)					
Married or cohabiting in United Kingdom	42/78 (53.8)	1.0 (0.5-1.7)	.95	NA	
Living apart relationship or marriage	44/78 (56.4)	1.1 (0.6-1.9)		NA	
Single or previously married	69/126 (54.8)	1 [Reference]		NA	
Religion (n = 216)					
Christian, all denominations	113/200 (56.5)	1 [Reference]	.54	NA	
Muslim	33/66 (50.0)	0.8 (0.4-1.3)		NA	
No or other religion	13/21 (61.9)	1.3 (0.5-3.2)		NA	
Sexual behavior (n = 275)					
Gay or bisexual	14/27 (51.9)	0.8 (0.4-1.8)	.65	NA	
Heterosexual	140/248 (56.5)	1 [Reference]		NA	
Attended emergency department or walk-in center in past 12 mo (n = 283)					
No	110/188 (58.5)	1 [Reference]	.56	NA	
Yes	17/28 (60.7)	1.3 (0.6-2.8)		NA	
Health service visit in last 12 mo (n = 216)^c^					
No	41/88 (46.6)	1 [Reference]	.003	1 [Reference]	.001
Yes	86/128 (67.2)	2.1 (1.3-3.4)		3.2 (1.6-6.4)	
Sexual health clinic attendance ever (n = 216)					
No	43/105 (41.0)	1 [Reference]	<.001	1 [Reference]	<.001
Yes	84/111 (75.7)	4.4 (2.6-7.4)		6.5 (3.1-13.7)	
STI diagnosis (n = 216)					
≤5 y	20/22 (90.9)	6.4 (2.2-19.0)	<.001	6.0 (1.2-29.8)	.005
>5 y	11/21 (52.4)	1.0 (0.4-2.3)		0.3 (0.1-0.9)	
Never	96/173 (55.5)	1 [Reference]		1 [Reference]	
No. of sexual partners in the past 12 mo (n = 281)					
None	17/44 (38.6)	1 [Reference]	.02	NA	
1	62/116 (53.4)	1.8 (0.9-3.7)		NA	
≥2	76/121 (62.8)	2.7 (1.3-5.6)		NA	
Used condom at last sex (n = 263)					
No	73/149 (49.0)	1 [Reference]	.006	NA	
Yes	75/114 (65.8)	2.0 (1.2-3.3)		NA	
Living with undiagnosed HIV (n = 208)					
No	115/199 (57.8)	1 [Reference]	.59	NA	
Yes	6/9 (66.7)	1.5 (0.4-6.0)		NA	

Abbreviations: aOR, adjusted odds ratio; NA, not applicable; OR, odds ratio; STI, sexually transmitted infection.

^a^ Adjusted for factors in model selection (age group, region of birth, education, health service visit in past 12 months, sexual health clinic attendance (ever), and STI diagnosis; n = 216).

^b^ Excludes people diagnosed with HIV longer than 5 years and item nonresponders.

^c^ Antenatal, general practitioner, emergency department, or NHS walk-in or outpatient appointment.

## Discussion

The findings from this community-based survey of black African men and women living and socializing in London in 2016 are simultaneously encouraging and concerning for policy makers and HIV program managers. We have found a relatively high uptake of HIV testing in the target population: more than two-thirds of respondents reported ever having had an HIV test, and more than half (53% of women and 51% of men) reported an HIV test in the past 5 years. These are higher levels of HIV testing within 5 years than previously reported (42%),^15,16^ and across the 2 Mayisha surveys, there was a marked increase in HIV testing within the past 5 years among men.

Recommendations from policy makers in the United Kingdom for the normalization of HIV testing initiated by health care professionals in a wide range of settings (including primary care),^25,26^ as well as London-wide^22^ and national^27^ HIV testing campaigns, may have contributed to the increase in HIV testing between the 2 Mayisha studies. While this finding is promising, the relatively low rates of HIV testing in this survey (in comparison with testing rates for men who have sex with men) are concerning given that 18 of 38 individuals (47.4%) who provided an HIV-positive specimen were reportedly unaware they were living with HIV. Nine of those living with undiagnosed HIV had tested within the past 5 years, indicating the persistence of barriers to HIV testing identified in the past (eg, HIV-related stigma and low perception of risk)^6,7,14,28^ and the need for an increase in repeated, frequent HIV testing among sexually active individuals. It is likely that the scale-up of HIV testing outside specialist sexual health services and devising effective testing interventions are needed to bring HIV testing rates among black African individuals in line with those among men who have sex with men.^25,26,29^

A substantial proportion of respondents had never been tested for HIV, had a serodiscordant or untested partner, and engaged in condomless sex. These figures are pertinent given the high HIV prevalence among black African heterosexuals in the United Kingdom. Furthermore, the high proportion of respondents who had received an STI diagnosis in the previous 5 years indicates the ongoing need for increased awareness of HIV acquisition in the United Kingdom. Reaching this group presents a major HIV- and STI- prevention challenge for public health specialists. To do so, HIV program managers need to implement strategies that target HIV testing and treatment at specific subgroups of black African individuals living in England. Multivariable analysis in this study indicated that women who had been in the United Kingdom for 11 to 20 years, women not accessing health services, men aged 18 to 24 years or 45 years and older, and men who did not continue in education past age 16 years are groups that might benefit from targeted HIV testing interventions and expansion of HIV testing modalities, including self-testing and self-sampling.

Despite the recent implementation of a large-scale trial of PrEP that includes black African individuals,^30^ few respondents in this survey reported knowledge of PrEP. This survey did not explore why PrEP knowledge was so low. However, HIV prevention programs should aim to increase awareness of PrEP among black African populations.

### Limitations

There are several limitations to this study. This survey used a convenience sample; therefore, selection bias may have affected the findings. We may have overestimated the prevalence of HIV testing because those who agreed to participate might have been more likely to have previously undergone HIV testing than those who declined. Although the response rate is lower than Mayisha II (56.5%),^21^ it is higher than surveys with this group (15.3% for the Bass Line 2009 survey)^15^ and reflects the difficulty of conducting sensitive face-to-face research in black African communities. While the large numbers of respondents who provided a specimen allowed us to estimate the proportion living with undiagnosed HIV, it is possible that the option of providing a sample deterred those who had not previously been tested. We aimed to limit social desirability bias by ensuring the survey was self-completed anonymously, and item completion was high, except for age, which was not reported by more than 10% of respondents. It is possible that social desirability bias led to an underestimate of serodifferent, condomless sex or of reporting STIs. The design of the study also excluded non-English or non-French speakers and those with very low levels of literacy.

The questionnaires used in the 2 surveys were almost identical, which allowed for comparison of outcomes. However, the surveys were conducted 12 years apart, and the venues used to recruit respondents—and the resulting sociodemographic makeup of the study samples—were different; therefore, differences in HIV testing prevalence are not directly comparable. Although the Mayisha 2016 sampling strategy used a robust mapping exercise based on UK census data, the final sample is not representative of the black African population in London, with black African individuals born outside Africa underrepresented.

We did not detect anti-HIV antibodies in 23.1% of respondents who reported living with diagnosed HIV, despite sufficient IgG concentrations. There are some possible explanations for this, including respondent error, laboratory error, and the possibility that antiretroviral therapy–induced long-term viral suppression reduces the production of anti-HIV antibodies—now well recognized with tests performed on oral fluid.^31^ The undiagnosed fraction detected in this London-based sample is substantially higher than national estimates for England (11% and 10% in black African heterosexual women and men, respectively)^32^; readers should interpret this finding with caution given that the study was not powered to estimate the prevalence of HIV or the undiagnosed fraction.

## Conclusions

Despite intensive efforts to increase HIV testing in African communities in London, almost half of respondents who supplied an HIV-positive oral fluid specimen self-reported that they were HIV negative or untested. This study provides some evidence that HIV testing among black African adults living in London has increased slightly in the past 12 years, but efforts must be enhanced to meet the HIV prevention needs of this group. Increasing access to initiated HIV testing initiated by health care professionals in primary care and other health care services, as well as access to HIV self-testing and self-sampling, may bolster HIV testing rates so that they are similar to those in other populations disproportionately represented in the HIV epidemic in England. High rates of serodifferent condomless last sex coupled with low knowledge of PrEP and undiagnosed infection in those tested within the past 5 years indicate that health promotion specialists should focus on highlighting the risks of HIV acquisition within the United Kingdom.

## Notes

Supplement. **eAppendix.** Supplemental Information **eTable 1.** Cut-off Ratio Results for Specimens From HIV Positive Patients **eTable 2.** Cut-off Ratio Results for Specimens From HIV Negative Volunteers **eTable 3.** Summary of False Negative Results **eReferences**
