# Supplementary material for: HIV Testing and Sexual Health Among Black African Men and Women in London, United Kingdom
Source: JAMA Netw Open. 2019 Mar 22;2(3):e190864. doi: 10.1001/jamanetworkopen.2019.0864 (PMC6583286; doi:10.1001/jamanetworkopen.2019.0864)
Supplement: Supplement. — eAppendix. Supplemental Information eTable 1. Cut-off Ratio Results for Specimens From HIV Positive Patients eTable 2. Cut-off Ratio Results for Specimens From HIV Negative Volunteers eTable 3. Summary of False Negative Results eReferences [file jamanetwopen-2-e190864-s001.pdf]

Supplementary Online Content

Fakoya I, Logan L, Ssanyu-Sseruma W, et al. HIV testing and sexual health among black African men and women in London, United Kingdom. *JAMA Netw Open*. 2019;2(3):e190864. doi:10.1001/jamanetworkopen.2019.0864

eAppendix. Supplemental Information

eTable 1. Cut-off Ratio Results for Specimens From HIV Positive Patients

eTable 2. Cut-off Ratio Results for Specimens From HIV Negative Volunteers

eTable 3. Summary of False Negative Results

eReferences

This supplementary material has been provided by the authors to give readers additional information about their work.

© 2019 Fakoya I et al. *JAMA Network Open.*

eAPPENDIX: SUPPLEMENTAL INFORMATION

Introduction

The Orasure oral fluid collection device has been used in a number of community-based surveys. This American product does not carry CE marking, which is used within the European Union to declare that a product complies with the essential requirements of the relevant European health, safety and environmental protection legislation. Although Orasure can be used for surveillance work, it can no longer be imported into the EU. The Orasure has been replaced by the Intercept® i2he™ device which does not contain any animal products and is available for purchase in the UK. It was developed and is primarily used for accurate testing for drugs for abuse (e.g., marijuana or cocaine) but can also be used for antibody testing. The Intercept 2 device uses a different transport media used to preserve the oral fluid from Orasure. Consequently, the standard assay used to test for HIV antibodies needed to be validated against the new Intercept 2 preservative. This report details the methods and findings of the validation exercise as well as findings from the Mayisha 2016 and Gay Men's Sexual Health (GMSHS) 2016 surveys.

Methods

The GACELISA is the standard assay that was used with previous Orasure devices. The validation exercise was undertaken in June 2016 as follows:

1. Oral fluid specimens were collected from 102 known HIV-positive patients at Hammersmith Broadway and St Stephens Centres, both clinics within the Chelsea and Westminster NHS Trust in London, United Kingdom.
2. Specimens were collected from 17 volunteers from Public Health England, Colindale. Volunteers were staff who had recently donated blood (via the workplace donation service) and were assumed to be HIV-negative.
3. All specimens were tested for sufficient immunoglobulin G (IgG). Assay sensitivity was determined using cut-offs for IgG antibody levels that conformed to surveillance (0.2mgs/l IgG) or clinical purposes (>1.0mgs/l IgG).

Results

Validation results

Of the eight specimens with OD/COCut Off/Optical Density/Cut Off (OD/COCO/OD) <1.0, one was rejected due to low IgG (concentration <0.2mg/l). Of the 17 known HIV negative specimens, all had a OD/CO/OD <1.0.

eTable 1: Cut-off Ratio results for specimens from HIV positive patients

OD/COCO/OD Range	Number of specimens
<1.0	8
1.0 - 10	60
>10	34
Grand Total	102

eTable 2: Cut-off ratio results for specimens from HIV negative volunteers

OD/COCO/OD Range	Number of specimens
<0.25	10
0.25 - 0.30	6
<0.3	1
Grand Total	17

© 2019 Fakoya I et al. *JAMA Network Open.*

Overall, after excluding specimens with insufficient IgG using surveillance standard cut-off values, sensitivity was 93% and using clinical cut-off values, sensitivity was 96%. IgG values on all HIV negative samples showed that all had sufficient IgG for surveillance purposes and no false positives were detected. These findings were sufficient to proceed with the surveys using the new Intercept® i2he™ device.

Mayisha 2016 and GMSHS 2016 survey results

All specimens were tested with GACELSIA, and those with an OD/CO of >10 were accepted as anti-HIV Positive. Specimens with an OD/CO of between 0.8 and 9.99 were re-tested, and if the mean of the two tests gave an OD/CO of >1.0 they were confirmed with Western Blot. For surveillance purposes such as these, the criteria for a specimen to be determined as anti-HIV detected by Western Blot is the presence of gp160 with or without other bands.

A total of 1208 oral fluid specimens were collected as part of the Mayisha and GMSHS surveys. Laboratory results were merged with questionnaire data using the unique identifier allocated in specimen acquisitions when completed packs were received.

Initial analysis revealed a higher than expected number of specimens testing negative when the individual had indicated via the questionnaire that they were HIV positive and therefore falsely negative. This assumes that data recorded on the questionnaires is accurate. There were more false negative specimens for the Mayisha survey compared to GMSHS, see below:

eTable3 Summary of false negative results

	**Mayisha**	**GMSHS**
Number of self-reported (SR) HIV positive participants who provided a specimen	32	42
Number of SR positive, specimen negatives	11	8
% false negative	34%	19%

Steps taken to investigate false negative results

1. *Total IgG concentration testing*

Total IgG concentration tests were initially conducted on a random sample of specimens. On discovery of the false negative issue, all specimens were tested for total IgG concentration.

IgG results for GMSHS 2016: 11/598 (1.8%) fell below 0.2mg/l cut off value

IgG Results for Mayisha 2016: 47/610 (7.7%) fell below 0.2mg/l cut off value

Significantly more low IgG specimens were collected in the Mayisha survey than in the GMSHS survey. Half of these specimens (21/42) were collected from outdoor sports events suggesting a specific issue with collection in this scenario.

One false negative from the GMSHS was eliminated due to the specimen having low IgG reducing the false negative rate to 17% (7/42). No correlation was found between low IgG and false negative results.

2. *Lowering the cut-off ratio*

From the two surveys, four specimens after repeat testing had an OD/CO in the range 0.8 to 1.0 (i.e. just below the cut-off of 1.0). One of these had been identified as a potential false negative by self-report and was tested by Western Blot. This specimen had trace reactivity HIV gp160 but would not normally have been considered as anti-HIV positive on the standard criteria applied. If this specimen is accepted as positive then the rate of false positives in the GMSHS is 14% (6/42).

3. *Transposition error*

© 2019 Fakoya I et al. *JAMA Network Open.*

Under further examination, two of the false negative results from the GMSHS occurred on the same plate and were adjacent to two strongly reactive specimens. This result was repeated after a second assay run. It is strongly suspected that a transposition error occurred but cannot be confirmed by returning to other identifiers as the specimens are anonymous.

By identifying the batch of 36 specimens within which these specimens were processed, it was possible to examine the data for the other results in the same batch. Taking into account the rest of the data from this batch, it appears likely that there was a transposition error. It is not possible to identify where within the batch the error occurred, so results from all 36 specimens in this run have been discarded (coded as error). This reduces the false negative rate for the GMSHS to 9.5% (4/42).

eReferences

Sadler KE, McGarrigle CA, Elam G, et al. Sexual behaviour and HIV infection in black-Africans in England: Results from the Mayisha II survey of sexual attitudes and lifestyles. *Sexually Transmitted Infections.* 2007;83(7):523-529.

Sexual behaviours, HIV testing, and the proportion of men at risk of transmitting and acquiring HIV in London, UK, 2000–13: a serial cross-sectional study Aghaizu, Adamma et al.The Lancet HIV , Volume 3 , Issue 9 , e431 - e440

© 2019 Fakoya I et al. *JAMA Network Open.*
